# Anesthetic Management of Resection of a Large Anterior Mediastinal Carcinoid Tumor

**DOI:** 10.7759/cureus.11688

**Published:** 2020-11-24

**Authors:** Geoffrey D Panjeton, Syed Hamaad Rahman, T. Everett Jones

**Affiliations:** 1 Anesthesiology, University of Florida, Gainesville, USA

**Keywords:** carcinoid tumor, general anesthesia, intratracheal intubation, mediastinal neoplasms, neuroendocrine tumors

## Abstract

This case report presents a 66-year-old man with chest pain and shortness of breath who had a 16 cm × 9 cm × 12-cm anterior mediastinal atypical carcinoid tumor with compression causing severe right ventricular outflow tract obstruction. We were consulted for anesthetic management of surgical resection of this tumor. Thoracic epidural, femoral, and radial arterial catheterizations, and femoral central venous access were performed with sedation. Upon ensuring adequate surgical site analgesia under thoracic epidural, chest incision was performed. Thereafter, induction and intubation were performed without complication. During intubation, fiberoptic bronchoscopy highlighted external compression of the left mainstem bronchus. The procedure was completed, and the patient was extubated in the operating room and transported to the intensive care unit in stable condition without complications.

## Introduction

Carcinoid tumors are a rare type of neuroendocrine tumor (NET) that arise from the gastrointestinal tract and bronchopulmonary systems with an annual incidence of two in 100,000 [1]. Carcinoid tumor survival correlates with staging, with a 71% five-year survival in patients with no metastases and a 38% five-year survival in patients with metastases [2]. Primary mediastinal NET is very rare with NETs of the thymus comprising less than 5% of all anterior mediastinal masses [3]. Primary NETs of the anterior mediastinum are highly aggressive and malignant in 82% of cases [3]. They often present in middle-aged (40- to 50-year-old) males (male/female ratio of three to one) [3]. We describe the anesthetic management considerations in a case of a massive anterior mediastinal carcinoid neoplasm.

## Case presentation

**Figure 1:**
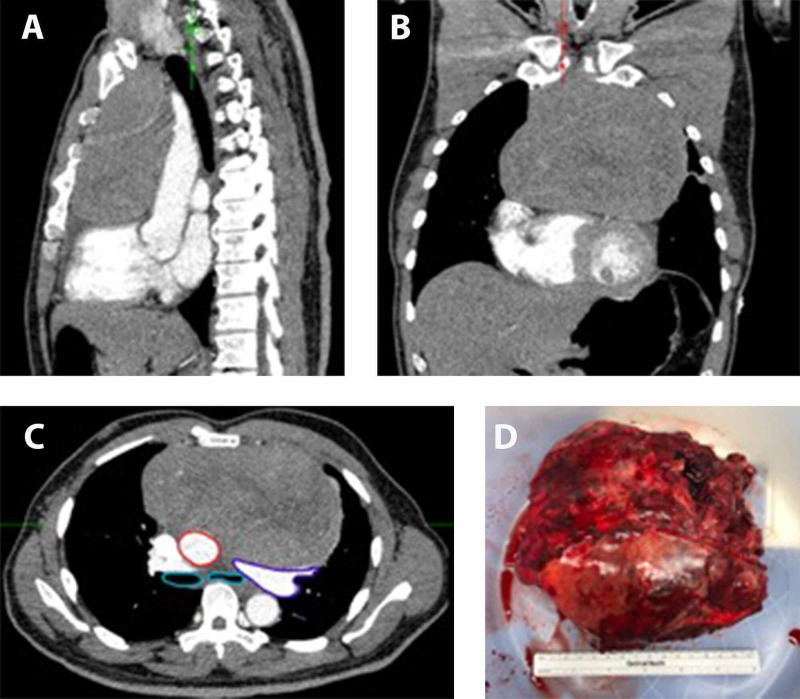
Radiographic and gross image of the mass Computed tomography imaging of the chest in (A) sagittal, (B) coronal, and (C) axial planes highlighting the extent of the mass. Panel C also highlights the extent of airway and vascular compression. The teal-colored circles reflect the bronchi and highlight left-sided bronchial compression. The structures outlined in red and purple represent the aorta and pulmonary artery, respectively, and the imaging highlights posterior displacement secondary to mass effect. (D) Photograph of the 16.1 cm × 9.2 cm × 12.3-cm mass after resection.

**Figure 2:**
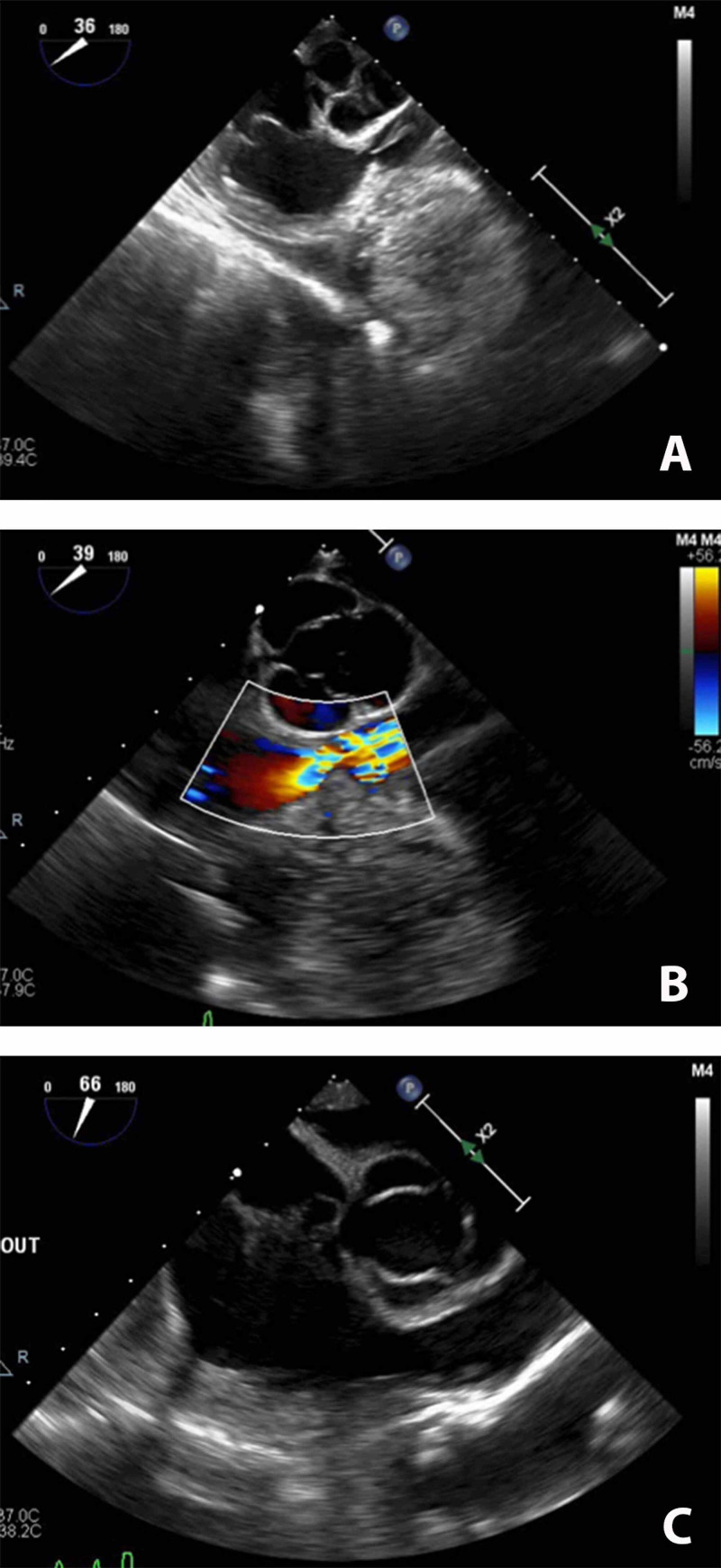
Transesophageal echocardiography imaging (A) Transesophageal echocardiography (TEE) imaging of moderate to severe right ventricular outflow tract obstruction with a pressure gradient of 55 mmHg. (B) TEE imaging of right ventricular outflow tract obstruction with color Doppler. (C) TEE imaging of the right ventricular outflow tract after resection of the mass.

A 66-year-old man with a past medical history of uncontrolled hypertension (requiring three agents with persistent systolic blood pressure readings above 180 mmHg), coronary artery disease with three prior cardiac stents placed, and acid reflux (worsening in the presence of the mass) presented to his outside physician with dysphagia, chest pain, and shortness of breath. Workup revealed a 16.1 cm × 9.2 cm × 12.3-cm anterior mediastinal mass (Figure 1) causing right ventricular outflow tract obstruction and displacement of the ascending aorta. This finding prompted a computed tomography-guided biopsy, which revealed a primary anterior mediastinal carcinoid tumor. Two-dimensional transthoracic echocardiography was performed, which corroborated findings of moderate to severe right ventricular outflow tract obstruction with a pressure gradient of 55 mmHg. Intraoperative transesophageal echocardiography highlighted this finding (Figure 2). His transthoracic echocardiogram was otherwise unremarkable. The patient was discussed in the Thoracic Oncology Tumor Board, and the recommendation was for surgical excision of the mass. Anesthesia was consulted for anesthetic management of the surgical tumor resection.

Anesthetic management proceeded with the placement of a thoracic epidural catheter at the T5-6 interspace via the institutionally preferred right paramedian approach in the preoperative holding area with no sedation required. A test dose of 3 mL lidocaine 1.5% with epinephrine 1:200,000 was administered with no hemodynamic changes. The patient was taken to the operating room, and procedural sedation was initiated with 1 mg of midazolam and a 0.8 mcg/kg/hr dexmedetomidine infusion. This medication regimen was chosen for anxiolysis and mild to moderate sedation while ensuring spontaneous ventilation. An additional benefit of this medication regimen is the minimal hemodynamic implications at this dose. The patient was positioned, prepared with antiseptic solution, and draped in the usual fashion. Radial arterial and femoral arterial and venous lines were placed under local anesthesia and conscious sedation. Following confirmation of adequate thoracic epidural anesthesia, a chest incision was made, and general anesthesia was induced with 2 mg of midazolam, 70 mg of ketamine, and 70 mg of propofol, maintaining spontaneous respiration while performing video laryngoscopy. Once a grade 1 view was obtained, muscle relaxant was administered, and a left-sided double-lumen endotracheal tube was placed and positioned appropriately with fiberoptic bronchoscopic guidance. Intermittent doses of phenylephrine were required to maintain hemodynamic stability throughout induction and surgical exposure and as such venoarterial extracorpeal membrane oxygenation cannulation and initiation were deemed unnecessary, although necessary equipment and staffing were on standby in the operating room.

Surgical resection was performed via clamshell incision at the fourth intercostal space. The tumor had invaded the pericardium, necessitating a pericardiectomy, as well as the left brachiocephalic vein, requiring tangential resection and repair. Further cephalad, the tumor had encased the left phrenic nerve, requiring clipping of the nerve proximal and distal to the tumor. Along the right thoracic inlet, the tumor had invaded the right internal mammary vessels, requiring clipping and ligation. Ultimately, the tumor was freed and removed from the field. Bilateral chest tubes were placed, the incision was closed in layers, and the sternum was closed with two sternal wire sutures. The patient was extubated in the operating room and transported to the intensive care unit in stable condition for further care. There were no intraoperative complications.

The postoperative pathology report reflected atypical carcinoid with positive margins as well as a pleural implant, denoting stage IV disease. His postoperative course was complicated by angioedema and emergent reintubation on postoperative day 2, presumably due to anaphylaxis associated with tramadol administration due to temporal relation to previously naïve medication administration. The incidence of tramadol-related angioedema is reported to be 1:1000-1:10,000 [4]. The patient developed ST segment elevation following the episode of angioedema along with elevated troponins, likely attributed to significant hypertension following treatment of angioedema. No further cardiac intervention was performed as echocardiography did not reveal consistently elevated cardiac enzymes or EKG changes. The patient was extubated once the airway edema resolved on postoperative day 4. He was then downgraded from intensive care on postoperative day 7 and discharged home in stable condition without further complication on postoperative day 8.

## Discussion

Patients with anterior mediastinal masses requiring surgical excision present a unique challenge for anesthesia providers. These patients are at risk for near-fatal airway obstruction and/or cardiovascular collapse with induction of anesthesia [5]. Therefore, it is of the utmost importance to perform an adequate preoperative evaluation focused on the extent of the mass and the clinical signs and symptoms manifested by the patient. The goals of an adequate preoperative evaluation of these patients is to (1) identify pathological diagnosis, (2) stage extent of malignancy, (3) identify a compromise of surrounding structures, and (4) evaluate for cardiorespiratory compromise [6]. Common signs and symptoms include chest pain or fullness, dyspnea, cough, sweats, superior vena cava obstruction, hoarseness, syncope, or dysphagia [5]. Most concerning among the signs and symptoms as related to increased perioperative risk are tracheal compression, orthopnea, or stridor when supine, indicative of increased risk of airway complications. Also, syncopal symptoms or pericardial effusions are concerning for increased risk of cardiovascular complications [5]. All patients should have a plain chest radiograph and computed tomography scan imaging to aid in planning for airway management and to reflect the extent of the mass [5]. Patients presenting with cardiovascular symptoms or those who are unreliable historians should also undergo transthoracic echocardiography to assess for cardiac, aortic, or pulmonary vascular compression [5]. 

Upon completion of this preoperative assessment, discussion between the anesthesia and procedural teams to determine whether the procedure can be performed under regional anesthesia versus general anesthesia is necessary. Should the procedure require general anesthesia, a thorough plan for induction, airway management, and adequate monitoring must be formed for safe care of the patient. Ultimately, the specifics of that plan will vary based on the clinical context and presentation of the patient. However, several considerations should be made related to the anatomical and physiological effects of an anterior mediastinal mass as it relates to general anesthesia (Table 1).

**Table 1:** Anesthetic considerations for patients with anterior mediastinal masses. ECMO: Extracorporeal membrane oxygenation; CPB, cardiopulmonary bypass.

Phase/Variable	Consideration
Preoperative	Review imaging to evaluate the extent of the mass effect on airway, respiration, cardiovascular structures, and circulation. Signs/symptoms of mass effect: activity tolerance, ability to lay flat, tachypnea, hoarseness, cough, facial/upper extremity edema, venous engorgement.
Position	Sitting minimizes the risk of hemodynamic instability; however, it can increase the difficulty of securing the airway and limit surgical exposure.
Respiration	Spontaneous respiration is optimal for maintenance of hemodynamic stability and airway patency. Controlled ventilation may be necessary to facilitate surgical exposure.
Induction	Ensure adequate vascular access. Inhalational induction can maintain spontaneous respiration; however, the airway should be secured quickly once deeper levels of sedation are reached. Titration of intravenous anesthetic agents may be appropriate, but this may have hemodynamic and airway consequences due to relaxing muscle tone and loss of spontaneous respiration. Muscle relaxation will cause loss of chest wall tone and may increase airway/cardiac chamber compression by mass effect. Consider the hemodynamic effect of the transition from spontaneous ventilation to mechanically controlled positive pressure ventilation.
Equipment	Double-lumen tube or microlaryngoscopy endotracheal tubes may be helpful to ensure appropriate ventilation can be maintained beyond any tracheal compression. Video laryngoscopy and fiberoptic bronchoscopy may assist in efficiently securing the airway, particularly in the presence of anatomical aberrances related to the mass. Rigid bronchoscopy and ventilation distal to the tumor compression may be helpful in life-threatening airway compression.
ECMO/CPB	Consider obtaining appropriate cardiopulmonary support access before induction and having necessary staff and equipment on standby in the operating room.
Intraoperative	Surgical complications: injury to airway structures, nerves, cardiac, and great vessels due to adhesions.
Postoperative	Assess readiness for extubation: consider bronchoscopy before extubation versus delayed extubation in the intensive care unit.

First, one must consider maintaining spontaneous respiration versus initiating controlled mechanical ventilation. Electing to maintain spontaneous respiration while securing the airway using an awake fiberoptic technique is often advocated and is advantageous in that it maintains the chest wall tone and the forces of active airway inspiration that may support a potentially compressed airway [5].

However, oversedation may result following administration of volatile anesthetics or intravenous anesthetic along with reduced lung volumes, relaxation of bronchial smooth muscle increasing the compressibility of the large airways, and obstructed respiration, which can further flatten a compressed trachea [5]. In the present case, central arterial and venous access was placed with spontaneous ventilation under moderate sedation and epidural anesthesia. Laryngoscopy was performed prior to induction, and once a favorable view was established, induction medications and paralytics were administered. This decision was made factoring in the patient’s hemodynamic stability with the supine position and advanced preparation for emergent cardiopulmonary support, if needed. Ultimately, as the patient had a favorable view with video laryngoscopy and remained hemodynamically stable, the decision was made to proceed with induction and muscular relaxation to facilitate endotracheal tube placement prior to securing the airway. Although in this patient endotracheal intubation performed in this manner was successful, it is important to recognize that an adequate view of the vocal cords leaves the potential for distal tracheal compression or possible collapse.

Related to induction techniques, careful consideration of hemodynamic support agents must be made prior to administration. Epinephrine may be beneficial as an inotropic agent, particularly if there is a concern for increased afterload related to left ventricular outflow tract obstruction and in case of emergent asystole during collapse. Norepinephrine, due to alpha receptor activity, and vasopressin may be less preferred due to concern for further increase in afterload and increased demand on the heart. These are general considerations and the decisions made for the present case; however, additional patient comorbidities must always be considered prior to deciding for any specific patient.

The next consideration involves positioning the patient to minimize risk of airway and cardiovascular complications. Compression from the anterior mediastinal mass can cause compression when supine that is improved with sitting upright. For this reason, many would advocate for maintaining the sitting position during induction to minimize the risk of respiratory and cardiovascular complications; however, this position can complicate airway management, and this challenge must be balanced with maintaining the patient’s hemodynamic stability. Due to the intricate relationship between positioning and mass-related cardiopulmonary obstruction, change in positioning should be considered early in the algorithm of managing hemodynamic or respiratory collapse during induction of anesthesia. Identifying positions that minimize the patient’s symptoms preoperatively can be helpful in guiding position changes during crises. Lateral, sitting, and reverse Trendelenburg positions can relieve obstruction; however, this may vary depending on the anatomy of the mass and related structures.

Finally, many would consider the option of femo-femoral cardiopulmonary bypass or venoarterial extracorporeal membranous oxygenation to avoid issues of gas exchange in patients with severe airway compromise, obstruction of the pulmonary artery, or as a rescue in the case of hemodynamic collapse with induction [7]. This could be facilitated by obtaining adequate access prior to induction with the assistance of local anesthesia and minimal conscious sedation as employed in the case description above. Advanced cannulation can provide central venous access for resuscitation if needed, recognizing the potential risks of exposing the patient to potential neurovascular injury. Slinger and Karsli argue that the time required to cannulate and establish adequate circulation and oxygenation once airway or cardiovascular collapse have already occurred would expose the patient to the potential for neurological injury even if they were able to be resuscitated, and as such establishing adequate cannulation and access prior to induction would be pertinent [5]. Preparations made in advance for emergent cardiopulmonary support must be coupled with discussions between surgical and anesthesia providers on thresholds at which point to initiate cardiopulmonary support. Collapse can manifest quickly, and an agreed-upon plan can save time during crisis situations. Sudden hypotension and respiratory obstruction manifested during induction of anesthesia prior to securing of the airway and adequate surgical exposure to manually relieve the obstruction should prompt consideration of emergent cardiopulmonary support. Specific hemodynamic and respiratory thresholds should be decided upon considering the patient’s additional underlying comorbidities. In the present case, a lower threshold mean arterial pressure of 50 mmHg and peripheral oxygen saturation of 85% or other signs of hemodynamic or pulmonary collapse were decided on as thresholds to consider emergent cardiopulmonary support.

Specific surgical considerations with anesthetic implications related to the extent of the mass include vascular and neural structure involvement. If the mass invades or compresses intrathoracic vascular structures, most notably causing superior vena cava obstruction, it would be prudent to consider large intravenous cannulation in the lower extremity to avoid complications related to obstruction of flow from mass effect or surgical resection [7]. Nerves essential to respiration, such as the recurrent laryngeal and phrenic nerves, may be involved in the mass extent and may require surgical resection [7]. In this circumstance, it may be appropriate to consider elective postoperative ventilation [7]. Close communication with the surgeon in both instances is essential as they have direct implications on anesthetic management and perioperative planning. In the present case, the patient did have resection of the phrenic nerve and despite concerns of spontaneous ventilatory ability, the patient was extubated without complication. This decision was made, given the patient’s favorable pulmonary function and airway anatomy following resection of the mass.

## Conclusions

This case highlights the intricate relationship between intrathoracic structures and cardiopulmonary stability. This patient had a large anterior mediastinal carcinoid tumor with known right ventricular outflow tract obstruction and displacement of the ascending aorta and later revealed endobronchial compression as well. Safe and effective anesthetic management of this patient requires addressing several of the factors mentioned above. Despite an extensive resection involving the brachiocephalic vein, phrenic nerve, and pericardium, the patient tolerated the procedure well without any intraoperative complications.
